# Relationship between outer retinal tubulation, retinal volume, and visual field in Bietti crystalline dystrophy

**DOI:** 10.1007/s00417-025-06742-8

**Published:** 2025-01-31

**Authors:** Yuka Kishi, Hanako O. Ikeda, Manabu Miyata, Shogo Numa, Takuro Kamei, Akitaka Tsujikawa

**Affiliations:** 1 Department of Ophthalmology and Visual Sciences, Kyoto University Graduate School of Medicine, Kyoto, Japan; 2 Department of Ophthalmology, Kobe City Nishi-Kobe Medical Center, Kobe, Japan; 3 Department of Ophthalmology, Osaka Medical and Pharmaceutical University, Osaka, Japan

**Keywords:** Outer retinal tubulation, Bietti crystalline dystrophy, Optical coherence tomography, Ophthalmology

## Abstract

**Purpose:**

To investigate the presence of tubulation in the outer nuclear layer of patients with Bietti crystalline dystrophy (BCD) using optical coherence tomography (OCT) and evaluate its relationship with visual field, visual field progression, and retinal volume.

**Methods:**

This retrospective cross-sectional study included 37 patients diagnosed with BCD who underwent spectral-domain OCT examination. OCT examinations and Humphrey visual field tests (10–2 program) were conducted. We performed correlation analyses to assess the correlation of the number of tubulations with the visual field parameters and retinal volume. We also compared the number and prevalence of tubulations in groups based on median values of the parameters. The primary outcome measure was the number and prevalence of tubulations.

**Results:**

The average age of the participants was 58.7 ± 9.6 years. The mean deviation (MD) value was −25.0 ± 9.0 decibels (dB). The MD slope value during an average follow-up period of 5.9 ± 3.8 years was −0.91 ± 1.02 dB/year. The number of tubulations tended to increase as the MD values worsened (*P* = 0.055, r = −0.33). Moreover, the number (*P* = 0.48) and prevalence (*P* = 0.42) of tubulations tended to be higher in the group with lower MD values. The number of tubulations decreased with worsening logarithmic minimum angle of resolution (logMAR) (*P* = 0.68, r = −0.07). The prevalence of tubulations was higher in the group with poorer logMAR (*P* = 0.068). We observed no significant correlations between the number of tubulations and the retinal outer, inner, or center volume (*P* = 0.46, r = −0.13; *P* = 0.76, r = 0.05; *P* = 0.47, r = 0.12, respectively). However, the prevalence of tubulations in the group with smaller retinal center volume was lower (*P* = 0.054).

**Conclusion:**

The number of tubulations correlated with the severity of visual field loss in patients with BCD; however, it did not correlate with visual field progression or retinal volume measurements. Further studies are needed to understand the development of tubulations and their implications for retinal atrophy in BCD.

**Supplementary Information:**

The online version contains supplementary material available at 10.1007/s00417-025-06742-8.

## Abstract (highlights)

**Key messages:**

*****What is known***:**

- Tubulations in the outer nuclear layer are known to develop in patients with Bietti crystalline dystrophy (BCD).

*****What is new***:**

- Tubulations are correlated with visual field defects during disease progression in patients with BCD.
- The number of tubulations increases in the early stages of the disease and tends to decrease with retinal atrophy, suggesting their potential use as indicators of retinal degeneration and disease progression.

**Supplementary Information:**

The online version contains supplementary material available at 10.1007/s00417-025-06742-8.

## Introduction

Bietti crystalline dystrophy (BCD) is a type of chorioretinal degeneration characterized by the presence of yellow-white crystalline deposits in the retina that are sometimes observed in the corneal limbus [1, 2]. BCD is inherited in an autosomal recessive manner and accounts for about 10% of all cases presenting with autosomal recessive retinal degeneration [2]. The prevalence of BCD is higher in Asian populations, especially the Japanese and Chinese populations [3]. Mutations in the cytochrome P450 family 4 subfamily V member 2 (*CYP4V2*) have been identified as a causative gene for BCD [4]. The onset of impairment occurs during the second or third decade of life [2], and progresses to visual field loss, poor night vision, and reduced visual acuity. Classifying the disease stages based on fundus autofluorescence, fundus photography, and optical coherence tomography (OCT) examinations has been proposed [5, 6]. Several studies have been conducted on gene therapy for *CYP4V2* [7–9] and drug therapy [10]; however, no treatment methods that can effectively suppress its progression have been established.

The crystalline deposits present in patients with BCD can be better appreciated as hyper-deposits on infrared images than fundus photographs [11–14]. OCT has been used to detect the disruption of retinal pigment epithelium (RPE) [11, 14] and the ellipsoid zone [15], as well as thinning of the outer nuclear layer (ONL). Recent studies have reported that higher measurements of foveolar thickness, choroidal thickness in the foveolar region, ellipsoid zone band length, and the outer retinal layers are associated with better visual acuity on average [16]. OCT examinations have also revealed the presence of circular structures, namely “tubulation,” in the ONL [5, 11, 12, 17–23] which are not commonly observed in other degenerative retinal diseases [11, 22]. These structures tend to disappear as the chorioretinal atrophy progresses [12]. Recent studies have suggested a relationship between the presence of outer retinal tubulation and the onset of RPE atrophy [18]; however, detailed reports regarding the specific conditions, stages, or progression states wherein tubulations are more likely to occur are lacking. Thus, further research is required to identify the factors contributing to the development of tubulation and its relationship with the progression and prognosis of retinal atrophy in patients with BCD.

This study examined the tubulations observed in the ONL of patients with BCD using OCT and investigated the conditions under which tubulations are observed in terms of visual field, visual field progression, and retinal volume. This is the first study to thoroughly investigate the relationship between outer retinal tubulation and visual field impairment.

## Methods

The Institutional Review Board and Ethics Committee of the Kyoto University Graduate School of Medicine approved this retrospective cross-sectional study. This study adhered to the tenets of the Declaration of Helsinki. All participants provided informed consent.

## Participants

This study included patients diagnosed with BCD who had undergone Spectral Domain (SD)-OCT examination between January 2005 and December 2022 at the Kyoto University Hospital. BCD was diagnosed based on the medical history and the findings of ophthalmologic examinations, including fundus examination, vision test, visual field test, and OCT examination, and confirmed via genetic testing for *CYP4V2*.

## Visual field test and OCT examination

We analyzed the mean deviation (MD) value of the 10–2 program (Swedish Interactive Thresholding Algorithm-standard) with Humphrey Field Analyzer (HFA) and excluded HFA tests with unreliable outcomes, such as a fixation loss of > 20%, a false-positive rate of > 15%, or a false-negative rate of > 33%. We subsequently calculated the MD slope using linear regression analysis with the least-squares method for patients with ≥ 2 HFA test results [24] at an interval of > 6 months, to evaluate the annual progress rate of the visual field.

The macula was scanned using SD-OCT (Spectralis HRA + OCT (Heidelberg Engineering, Heidelberg, Germany)). An investigator (Y.K.) counted the number of tubulations on the horizontal and vertical 30°-scans crossing the fovea. The total number of horizontal and vertical scans was used as the number of tubulation for the analysis. To analyze tubulation distribution, horizontal and vertical scans were used to measure the number of tubulations present in 13 regions: the center region, the inner and outer circles of the Early Treatment Diabetic Retinopathy Study (ETDRS) grid (subdivided into temporal, inferior, nasal, and superior quadrants), and the area outside the ETDRS grid (Supplementary Fig. S1). Additionally, the proportion of tubulations in each of these regions was calculated. The number and proportion of tubulations were then divided by the area of each region to obtain area-adjusted values. A senior grader (H.I.) confirmed the accuracy of the counts. We measured the retinal volume using the built-in software in the Heidelberg Engineering OCT system and evaluated the retinal volume using the ETDRS grid. The area between 3 and 6 mm in diameter corresponded to the outer volume, the area between 1 and 3 mm in diameter corresponded to the inner volume, and the central area of 1 mm diameter corresponded to the center volume (Supplementary Fig. S1).

Staging of disease progression was performed using fundus autofluorescence imaging and OCT images based on a previously reported method [6].

## Selection of data

We investigated the availability of the HFA data and retinal volume data of OCT within the 1-year period before and after the OCT examination of the horizontal and vertical macular cross-scans. We selected the HFA and retinal volume data of OCT performed within 1 year before and after the examination with the highest number of data points if multiple OCT examinations of the horizontal and vertical macular cross scans were available. We used the data obtained closer to the OCT examination if more than one data point was present within 1 year before and after the OCT examination. We used the oldest data if the number of data points was the same. We extracted the age, visual acuity (VA), and MD values based on the date of the OCT examination for statistical analysis and converted the decimal visual acuity to the logarithmic minimum angle of resolution (logMAR). The VA was converted to logMAR using the following formula: a VA of counting finger corresponded to a logMAR value of 2.6, a VA of hand motion corresponded to a logMAR value of 2.9, a VA of light perception corresponded to a logMAR value of 3.2, and a VA of no light perception corresponded to a logMAR value of 3.4 [25]. All patients had data for both eyes. The data from the left and right eyes showed a high level of correlation; therefore, we performed statistical analyses using the data from the right eye.

## Statistical analysis

We conducted a correlation analysis for each of the following parameters and determined the correlation coefficients: MD, MD slope, logMAR, retinal outer volume, retinal inner volume, retinal center volume, and tubulation number. We calculated the median values of MD, MD slope, logMAR, retinal outer volume, retinal inner volume, and retinal center volume, and categorized the corresponding data into two groups: those with values smaller than the median and those with values equal to or greater than the median. We used the Wilcoxon signed-rank test to compare the number of tubulations between the groups. We used the chi-squared test to investigate the presence of tubulation and analyze the prevalence of tubulation in each group. We also performed multivariate analysis using a mixed-effects model to investigate parameters related to the number of tubulations and divided the data into two groups based on the presence or absence of tubulations. We also compared the respective parameter values and used a t-test to examine the retinal outer and inner volumes. We used the Wilcoxon signed-rank test to examine the remaining parameters. We performed multivariate analysis using a mixed-effects model with MD, logMAR, and retinal center volume as factors. These parameters were selected based on their *P*-values (< 0.1) in the correlation analysis with the number of tubulations, comparison of the proportion of eyes with tubulations between groups stratified by these parameters, and comparison of each parameter between eyes with and without tubulations. Statistical significance was set at *P* < 0.05. We performed all statistical analyses using JMP Pro software (v. 16.2.0; SAS Institute Japan Ltd., Tokyo, Japan). Data are presented as means ± standard deviations, where applicable.

## Results

### Participants

This study included 37 patients. Table 1 summarizes the patient characteristics. The mean age of the patients was 58.7 ± 9.6 years, and the mean MD value of the HFA10-2 tests was −25.0 ± 9.0 decibels (dB) (Table 1). Regarding *CYP4V2* mutations, 20 patients exhibited a homozygous c.802-8_810delinsGC mutation, one patient had a homozygous c.518T>G mutation, and the remaining patients showed compound heterozygous mutations (Supplementary Table S1). Twenty-five eyes underwent ≥ 2 HFA10-2 tests during the observation period of > 6 months. The observation period ranged from 0.77 to 14.05 years, with a mean duration of 5.9 ± 3.8 years. The mean MD slope was −0.91 ± 1.02 dB/year (Table 2).

**Table 1:** Characteristics of the patients and eyes at time of data acquisition

	N	Mean ± SD	Range (Median)
Age (year)	37 patients	58.7 ± 9.6	37 to 77 (60)
Sex, N (%)	37 patients		
Male		13 (35)	
Female		24 (65)	
Visual acuity, LogMAR	37 eyes	0.36 ± 0.62	−0.18 to 2.60 (0.10)
MD (dB)	34 eyes	−25.0 ± 9.0	−33.62 to −1.25 (−28.56)
Retinal volume (mm^3^)	36 eyes		
Retinal outer volume		1.46 ± 0.21	0.38 to 1.93 (1.46)
Retinal inner volume		0.49 ± 0.07	0.24 to 0.67 (0.49)
Retinal center volume		0.22 ± 0.05	0.08 to 0.30 (0.24)

LogMAR, logarithmic minimum angle of resolution; MD, mean deviation of the Humphrey Field Analyzer 10–2 program; dB, decibel; SD, standard deviation

**Table 2:** MD slope during the follow-up period

	Mean ± SD	Range (Median)
N = 25 eyes		
Follow-up period (years)	5.9 ± 3.8	0.77 to 14.05 (6.46)
MD slope (dB/year)	−0.91 ± 1.02	−4.15 to 0.20 (−0.60)

MD, mean deviation of the Humphrey Field Analyzer 10–2 program; dB, decibel; SD, standard deviation

**Fig. 1:**
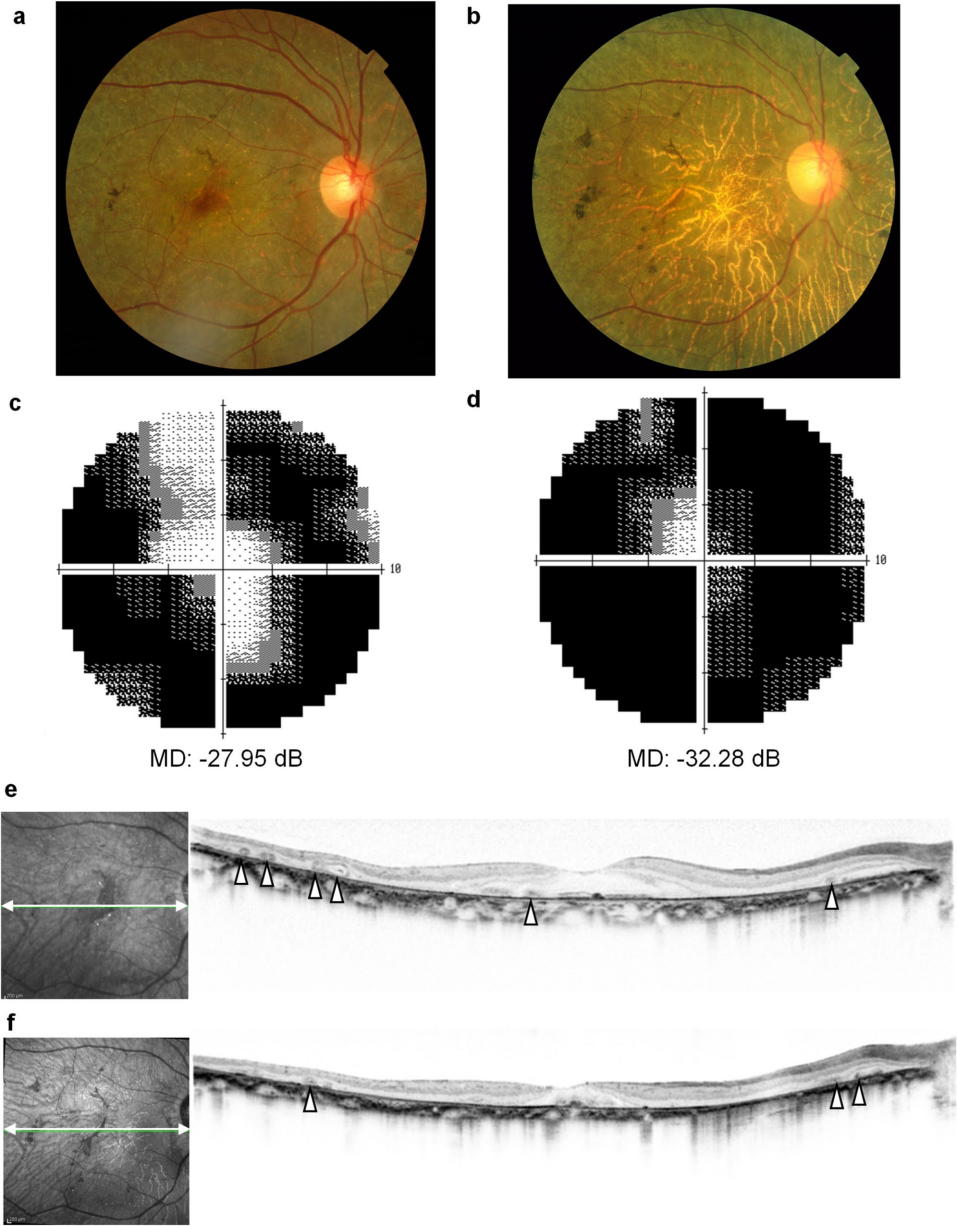
Progression of Bietti crystalline dystrophy. (**a**) and (**b**) Fundus photographs of a patient with Bietti crystalline dystrophy (BCD). (**c**) and (**d**) Humphrey visual field test results with the 10–2 program and the mean deviation (MD) values. dB: decibel. (**e**) and (**f**) Horizontal images of optical coherence tomography acquired at the first visit (**a**, **c**, **e**, when the patient was in her 50 s) and 14 years later (**b**, **d**, **f**, when the patient was in her 60 s). The arrowheads in (**e**) indicate tubulations in the outer nuclear layer

**Fig. 2:**
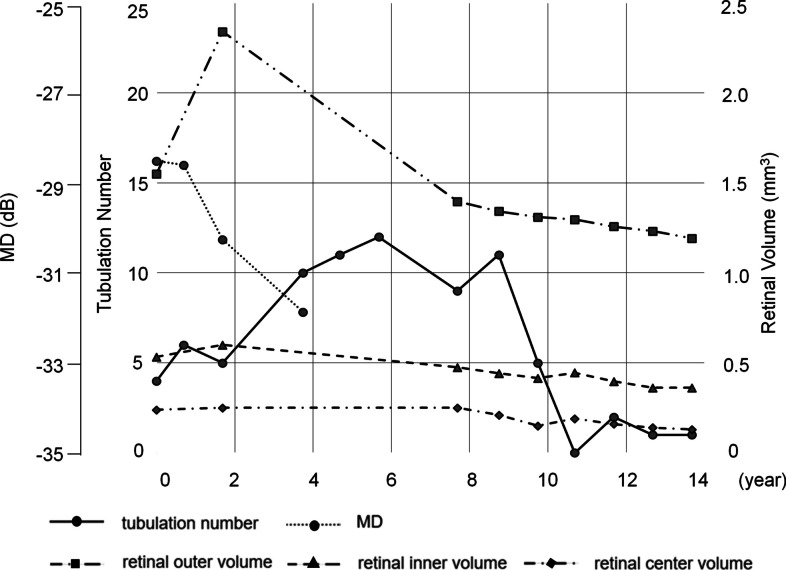
Natural course of Bietti crystalline dystrophy. The X-axis shows the year since the first visit (when the patient was in his 60 s). The Y-axis shows the mean deviation (MD) values for the Humphrey visual field test with the 10–2 program, tubulation number, retinal outer volume, retinal inner volume, and retinal center volume (see Supplemental Fig. S1). dB: decibel

Figure 1 presents the fundus photographs, results of the HFA10-2 visual field test, and OCT images of a typical patient with BCD acquired at the first visit and 14 years later. Retinal degeneration continued to progress, and the degenerative area expanded during the 14-year observation period. The number of yellow-white crystals at the fundus observed at baseline had decreased after 14 years (Fig. 1a and 1b). The findings of the HFA10-2 visual field test revealed the progression of visual field loss from −27.95 to −32.28 dB in the MD value over a period of 14 years (Fig. 1c and 1d). The calculated MD slope was −0.43 dB/year. The OCT images revealed degeneration of the RPE. Round-shaped structures, namely tubulations, were observed in the ONL at baseline (arrowheads in Fig. 1e). Thinning of the outer retinal layers, particularly in regions further from the fovea, had progressed, and the number of tubulations had decreased after 14 years (Fig. 1f). Figure 2 presents a typical example of the natural course of the number of tubulations, MD value, and retinal volume. The MD value deteriorated gradually, and the test was discontinued midway as the sensitivity was near 0 dB at almost all test points in the HFA 10–2 test. The number of tubulations increased over time, remained high for a period, then decreased gradually. The retinal volume started decreasing from the outer region and decreased in the central region during the latter half of the observation period. The number of tubulations was higher during the period when the outer and inner retinal volumes were decreasing. Therefore, we investigated the relationship between the number of tubulations and MD values as well as the retinal volume.

### Correlations between the number of tubulations and other parameters

**Fig. 3:**
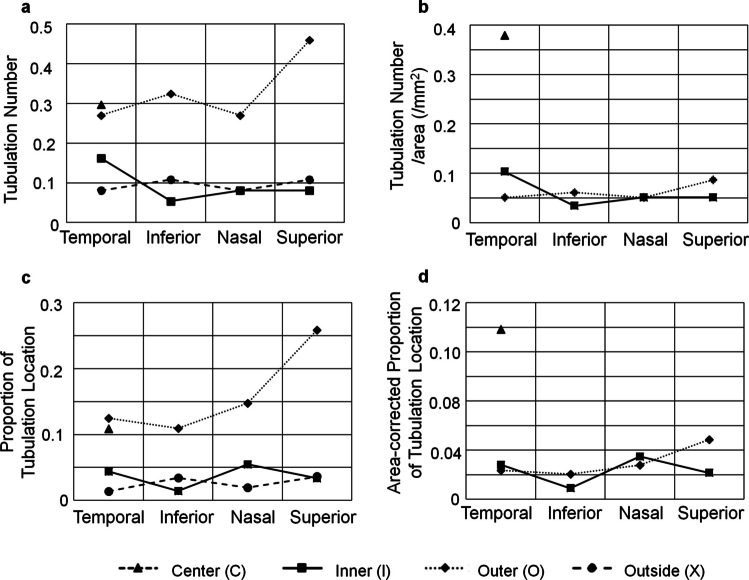
Analysis of tubulation distribution. The number of tubulations (**a**), number of tubulations normalized by area (tubulations per unit area) (**b**), proportion of regions containing tubulations (**c**), and proportion of regions containing tubulations normalized by area (area-adjusted tubulation proportion) (**d**) are shown. Each region within and outside the Early Treatment Diabetic Retinopathy Study (ETDRS) grid is shown in Supplementary Fig. S1

First, we examined the distribution of tubulations across different regions. The analysis revealed that the number and proportion of tubulations were higher in the center and outer regions of the ETDRS grid (Fig. 3a, c). However, given the differences in the area of each region, area-adjusted results showed that the number and proportion of tubulations were highest in the center region of the ETDRS grid, with no significant difference between the inner and outer regions (Fig. 3b, d). Additionally, there were no notable differences in the distribution of tubulations among the temporal, inferior, nasal, and superior quadrants.

**Fig. 4:**
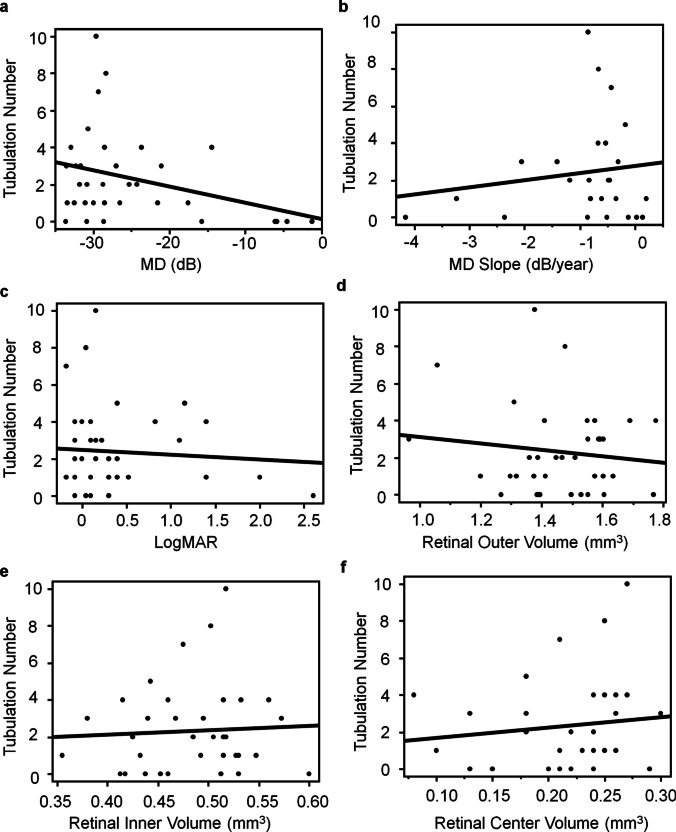
Correlations between the number of tubulations and visual field data and retinal volume. (**a**) Scatterplots and linear regression of the number of tubulations with the mean deviation (MD) value of Humphrey field analyzer 10–2 (**a**, N = 34, *P* = 0.055, r = −0.33), MD slope (**b**, N = 25, *P* = 0.48, r = 0.15), logarithmic minimum angle of resolution (logMAR) (**c**, N = 37, *P* = 0.68, r = −0.07), retinal outer volume (**d**, N = 37, *P* = 0.46, r = −0.13), retinal inner volume (**e**, N = 37,* P* = 0.76, r = 0.05), and retinal center volume (**f**, N = 37, *P* = 0.47, r = 0.12)

The number of tubulations showed a negative correlation with the MD values (Fig. 4a, *P* = 0.055, r = −0.33). In contrast, the number of tubulations did not show any clear correlations with the MD slope or logMAR (Fig. 4b, *P* = 0.48, r = 0.15, Fig. 4c, *P* = 0.68, r = −0.07). The number of tubulations tended to increase as the retinal outer volume decreased; however, no significant correlation was observed (Fig. 4d, *P* = 0.46, r = −0.13). Similarly, the number of tubulations did not show any clear correlations with the retinal inner volume or the retinal center volume (Fig. 4e, *P* = 0.76, r = 0.05, Fig. 4f, *P* = 0.47, r = 0.12).

Next, the disease stage was evaluated and the number of tubulations was compared among the stages (Table 3); there were no differences among the stages (*P* = 0.29).

**Table 3:** Staging by fundus autofluorescence and number of tubulations

Stage*	N	Number of tubulations	
	(eyes)	Mean ± SD	Range
2A	2	0 ± 0	0–0
2B	8	2.1 ± 2.5	0–7
3A	20	2.9 ± 2.3	0–10
3B	7	1.9 ± 1.8	0–4

*Staging by Fundus Autofluorescence was performed according to the study by Li et al. [6]

### Correlations between the prevalence of tubulation and other parameters

**Fig. 5:**
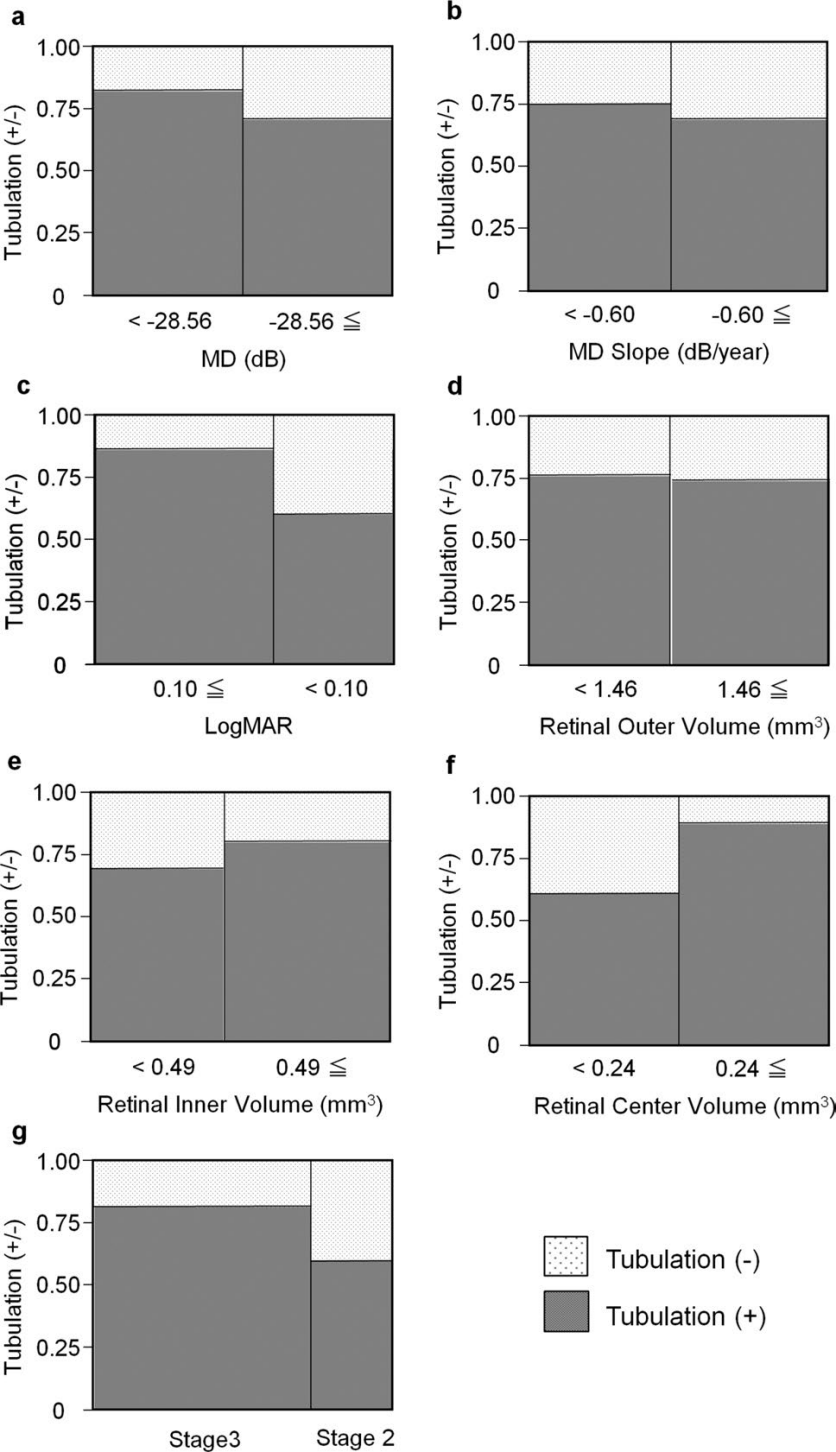
Relationship between the presence or absence of tubulations and visual field data and retinal volume. Mean deviation (MD), MD slope, logarithmic minimum angle of resolution (logMAR), retinal outer volume, retinal inner volume, and retinal center volume data are categorized into two groups: those with values smaller than the median and those with values equal to or greater than the median. Chi-squared tests were performed to evaluate the proportion of tubulation present in each group of MD value of Humphrey visual field analyzer 10–2 (**a**, N = 34 eyes,* P* = 0.42), MD slope (**b**, N = 25 eyes, *P* = 0.75), logMAR (**c**, N = 37, *P* = 0.068), retinal outer volume (**d**, N = 37 eyes, *P* = 0.85), retinal inner volume (**e**, N = 37 eyes, *P* = 0.44), retinal center volume (**f**, N = 37 eyes, *P* = 0.054). (**g**) Comparison of the proportion of tubulation presence between Stages 2 and 3 [6]. Dotted boxes: eyes without tubulation, gray boxes: eyes with tubulation

We calculated the median values of the MD value, MD slope, logMAR, retinal outer volume, retinal inner volume, and retinal center volume (Tables 1 and 2) and categorized the corresponding data into two groups: those with values smaller than the median and those with values equal to or greater than the median. The mean number of tubulations in the eyes with an MD value of less than −28.56 dB (median value of the included eyes) was 2.6 ± 2.7, which was higher than that in the eyes with an MD value of −28.56 dB or greater (2.0 ± 2.2; *P* = 0.48); however, the difference was not statistically significant. Similarly, comparison of the proportion of eyes with and without tubulations between the two groups revealed that the proportion of eyes with tubulations in the group with poorer MD values was higher than that in the group with better MD values; however, the difference was not statistically significant (Fig. 5a, 0.82 vs. 0.71, *P* = 0.42). The mean number of tubulations in the eyes with an MD slope poorer than −0.60 dB/year was 2.8 ± 3.2, which was higher than that in the eyes with an MD slope of −0.60 dB/year or better (2.0 ± 2.2; *P* = 0.58). Comparison of the proportion of eyes with and without tubulations between the two groups revealed that the proportion of eyes with tubulations in the group with the poorer MD slope was not significantly different from that in the group with a better MD slope (Fig. 5b, 0.75 vs. 0.69, *P* = 0.75). The mean number of tubulations in the eyes with a logMAR value of < 0.10 was 2.1 ± 2.6, which was smaller than that in the group with a higher logMAR value (2.5 ± 2.3; *P* = 0.32). The proportion of eyes with tabulations in the group with a higher logMAR value was also higher than that in the group with a lower logMAR value (Fig. 5c, 0.60 vs. 0.87, *P* = 0.068).

The mean number of tubulations in the eyes with a retinal outer volume of < 1.46 mm^3^ and ≥ 1.46 mm^3^ was 2.4 ± 2.8 and 2.3 ± 2.1, respectively (*P* = 0.78). The proportion of eyes with tubulations in the group with a smaller retinal outer volume was almost identical to that in the group with a larger retinal outer volume (Fig. 5d, 0.76 vs. 0.74, *P* = 0.85). The mean number of tubulations in the eyes with a retinal inner volume of < 0.49 mm^3^ and > 0.49 mm^3^ did not differ significantly (2.2 ± 2.1 vs. 2.4 ± 2.6, respectively, *P* = 0.96). Comparison of the proportion of eyes with and without tubulations between the two groups revealed that the proportion of eyes with tubulations in the group with a smaller retinal inner volume did not differ significantly from that in the group with a larger retinal inner volume (Fig. 5e, 0.69 vs. 0.80, *P* = 0.44). In contrast, the mean number of tubulations in the eyes with a retinal center volume of < 0.24 mm^3^ and ≥ 0.24 mm^3^ was 1.8 ± 2.0 and 2.8 ± 2.6, respectively; however, the difference was not statistically significant (*P* = 0.15). Similarly, comparison of the proportion of eyes with and without tubulations between the two groups revealed that the proportion of eyes with tubulations in the group with a smaller retinal center volume was lower than that in the group with a larger retinal center volume (Fig. 5f, 0.61 vs. 0.89, *P* = 0.054).

The mean number of tubulations in the eyes at stage 3A and 3B (severe stages) was 2.6 ± 2.2, which was higher than that in the eyes at stage 2A and 2B (1.7 ± 2.4; *P* = 0.30). Comparison of the proportion of eyes with and without tubulations between the two stages revealed that the proportion of eyes with tubulations in stage 3 was higher than that in stage 2, though there was no statistical difference (Fig. 5g, 0.81 vs. 0.60, *P* = 0.18).

Table 4 presents the mean values of each parameter in the eyes with and without tubulations. The mean of the MD values in the eyes with and without tubulations was −27.9 ± 4.9 dB and −15.8 ± 13.3 dB, respectively (*P* = 0.059).

**Table 4:** Mean values of the parameters of the eyes with and without tubulation

	Tubulation ( +)			Tubulation (-)			P*
	N	Mean ± SD	Range	N	Mean ± SD	Range	
Age (year)	28	58.9 ± 8.8	42 to 77	9	58.2 ± 12.9	37 to 76	0.99
Visual acuity, LogMAR	28	0.38 ± 0.56	−0.18 to 2.0	9	0.31 ± 0.87	−0.08 to 2.6	0.19
MD (dB)	26	−27.9 ± 4.9	−33.5 to −14.4	8	−15.8 ± 13.3	−33.6 to −1.3	0.059
MD slope (dB/y)	18	−0.82 ± 0.78	−3.23 to 0.20	7	−1.12 ± 1.59	−4.15 to 0.14	0.83
Retinal volume (mm^3^)							
Retinal outer volume	27	1.45 ± 0.18	0.97 to 1.78	9	1.49 ± 0.15	1.27 to 1.77	0.57
Retinal inner volume	27	0.49 ± 0.05	0.36 to 0.57	9	0.48 ± 0.06	0.41 to 0.60	0.92
Retinal center volume	27	0.22 ± 0.05	0.08 to 0.30	9	0.21 ± 0.05	0.13 to 0.29	0.23

LogMAR, logarithmic minimum angle of resolution; MD, mean deviation of the Humphrey Field Analyzer 10–2 program; dB, decibel; SD, standard deviation

*t-test for the retinal outer and inner volumes, the Wilcoxon signed-rank test for others

### Multivariate analysis of tubulation numbers

Multivariate analysis performed using MD values, logMAR values, and the retinal center volume as potential factors that may be associated with the number of tubulations revealed that MD values had a significant impact on the number of tubulations (Table 5).

**Table 5:** Factors affecting the number of tubulation

	B	95% CI	β	*P*
MD	−0.11	−0.20 to −0.02	−0.42	0.02
LogMAR	−1.15	−2.58 to 0.28	−0.29	0.11
Retinal center volume	5.18	−12.06 to 22.42	0.10	0.54

CI, confidence interval; LogMAR, logarithmic minimum angle of resolution; MD, mean deviation of the Humphrey Field Analyzer 10–2 program

## Discussion

We investigated the characteristics and correlations of outer retinal tubulations in patients with BCD. Examining the correlations between the number of tubulations and various parameters revealed an increasing trend for the number of tubulations with the worsening of the MD values of the HFA 10–2 test. However, we observed no significant correlation between the number of tubulations and any of the parameters (Fig. 4). Analysis of the prevalence of tubulations in relation to other parameters revealed that the proportion of eyes with tubulations tended to be higher in groups with poorer MD and logMAR values. Moreover, we observed that the prevalence of tubulation was lower in the group with a smaller retinal center volume (Fig. 5). These findings indicate that tubulation is more likely to be observed in patients with advanced disease stages. However, tubulations are observed less frequently as retinal degeneration progresses closer to the fovea.

Tubulation is commonly observed in areas showing active degeneration and is rarely observed in areas with an intact RPE layer [23]. Thus, the presence of tubulations may serve as a sign of RPE atrophy [18] and is associated with photoreceptor or RPE injury [13]. Tubulations develop via the rearrangement of the photoreceptor layer and the formation of new connections [13, 21]. RPE degeneration precedes photoreceptor degeneration in patients with BCD, thereby compromising visual function [26, 27]. The findings of our study indicate the presence of a correlation between RPE degeneration and prevalence of tubulations, as well as thinner central retina and lower occurrence of tubulation. As previously reported [5], tubulations were observed in both stage 2 and stage 3, with the more severe stage 3 exhibiting a higher number and a greater proportion of areas with tubulations. These findings provide insight into the role of tubulations in patients with BCD. The annual changes in the MD in the patients with BCD included in our study was −0.91 dB/year. Previous studies have reported that the annual changes in the MD of patients with retinitis pigmentosa (RP) ranges from −0.46 to −0.23 dB/year [24, 28, 29]. Rod photoreceptors are considered to be the main target of the disease in most patients with RP [26]. However, the cone and rod photoreceptors are equally affected in patients with RPE degeneration [23]. Therefore, the rate of MD deterioration in patients with BCD may be faster than that in patients with RP. However, there may be variations in the baseline MD values and age between the two groups, and the small sample size of our study warrants further investigation.

This study has certain limitations. First, we counted the number of tubulations manually on two cross-sections of OCT images. However, it is possible that there were tubulations present beyond the selected cross-sections that we did not count. Although volume scans were performed in many cases, it remains difficult to clearly determine how the tubulations connect across adjacent images. Therefore, in this study, we substituted the total number of tubulations with the number observed on horizontal and vertical OCT scans, though there are no other reported studies that evaluated tubulations using horizontal and vertical scans alone, which further highlights the limitations of this approach. Second, disease progression was assessed solely by the progression of the visual field (MD slope); however, it would have been meaningful to compare this with changes in other imaging modalities, such as the degeneration areas observed in autofluorescence fundus imaging (dark areas). Unfortunately, owing to the retrospective nature of this study, regular autofluorescence imaging was not available for a significant number of patients, which limited our ability to include these data in the analysis. Third, we examined the data of the same patient acquired on different days up to 2 years apart. It may be more suitable to use data acquired at the same time for analysis, considering disease progression. Finally, the sample size of this study was relatively small at 37 eyes. The small sample size may be reflective of the rarity of BCD; however, it poses challenges in conducting extensive and prolonged observations on a larger patient cohort. Nonetheless, these limitations should be considered in the context of the inherent difficulties associated with evaluating a rare disease, such as BCD, and the resulting limited availability of a larger patient population for long-term follow-up.

In conclusion, our study revealed the correlation between outer retinal tubulations and visual field defects during disease progression in patients with BCD. The number of tubulations increases during the early stages of the disease; however, it tends to decrease as retinal atrophy progresses. They may serve as an indicator of retinal degeneration and disease progression. However, further studies must be conducted to understand their significance in monitoring BCD. Our findings emphasize the clinical relevance of tubulations and the importance of ongoing research for formulating new disease management strategies.

## Supplementary Information

Below is the link to the electronic supplementary material.

Supplementary file1 (PDF 168 KB)

## Data Availability

The data generated during the current study are available from the corresponding author on reasonable request.
