# Supplementary material for: Relationship between outer retinal tubulation, retinal volume, and visual field in Bietti crystalline dystrophy
Source: Graefes Arch Clin Exp Ophthalmol. 2025 Jan 31;263(4):993–1003. doi: 10.1007/s00417-025-06742-8 (PMC12095397; doi:10.1007/s00417-025-06742-8)
Supplement: Supplementary file 1 — Supplementary file1 (PDF 168 KB) [file 417_2025_6742_MOESM1_ESM.pdf]

Supplementary Table S1. ***CYP4V2*** **mutations in patients**

N	Mutation 1	Mutation 2
20	c.802-8_810delinsGC	c.802-8_810delinsGC
1	c.518T>G	c.518T>G
1	c.802-8_810delinsGC	c.219T>A
1	c.802-8_810delinsGC	c.327+1G>A
1	c.802-8_810delinsGC	c.1020G>A
2	c.802-8_810delinsGC	c.1090G>A
2	c.802-8_810delinsGC	c.1091-2A>G
1	c.802-8_810delinsGC	c.1199G>A
2	c.802-8_810delinsGC	c.1226-6_1235del
2	c.802-8_810delinsGC	c.518T>G
1	c.802-8_810delinsGC	c.1378T>C
1	c.1091-2A>G	c.992A>C

In two patients, *CYP4V2* mutations were confirmed through genetic testing conducted at other institutions; however, the specific details of the mutations are unknown.

Supplementary Figure S1

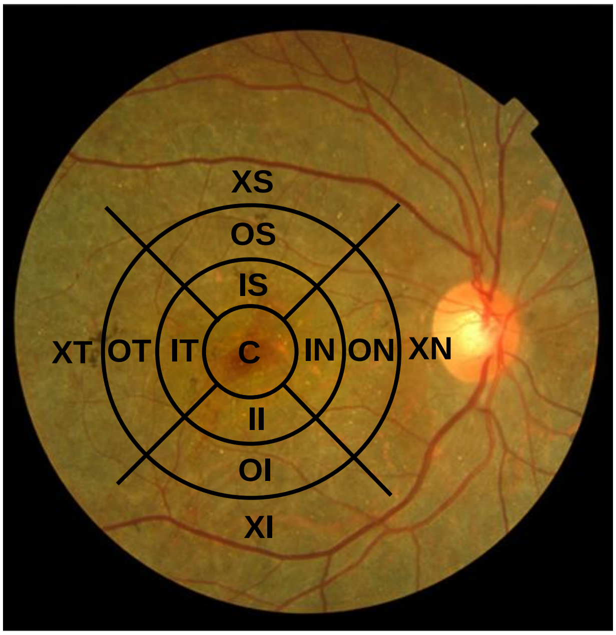

C : Center
I : Inner
O : Outer
X : Outside

T : Temporal
I : Inferior
N : Nasal
S : Superior

Supplemental Figure S1.
Measurement of the tubulation number and retinal volume.
The distribution of tubulations and retinal volume was evaluated using the Early Treatment Diabetic Retinopathy Study (ETDRS) grid. Retinal volume was measured in the following regions: the area between 3 mm and 6 mm in diameter corresponding to the outer volume (labeled as O), the area between 1 mm and 3 mm in diameter corresponding to the inner volume (labeled as I), and the central area within 1 mm in diameter corresponding to the center volume (labeled as C). Additionally, X indicates the area outside the 6 mm circle; T, I, N, and S denote temporal, inferior, nasal, and superior quadrants, respectively.
